# Supplementary material for: Rehabilitation after Hip Fracture Surgery: A Survey on Italian Physiotherapists’ Knowledge and Adherence to Evidence-Based Practice
Source: Healthcare (Basel). 2023 Mar 8;11(6):799. doi: 10.3390/healthcare11060799 (PMC10048225; doi:10.3390/healthcare11060799)
Supplement: Supplementary file 1 [file healthcare-11-00799-s001.zip › healthcare-2244534-supplementary.pdf]

healthcare

MDPI

Questions	Answers
1. In quale regione lavori	
2. Genere	Maschio<br>Femmina
3. Quanti anni hai?	
4. Da quanti anni svolgi la professione di fisioterapista?	
5. Quale è stata la tua formazione post-lauream	Corsi ECM<br>Master di I livello<br>Laurea Magistrale<br>Dottorato di Ricerca
6. Sei in possesso del titolo OMPT?	Si<br>No<br>Studente del Master
7. In quale setting si svolge principalmente la tua attività clinica?	Ospedale Pubblico<br>Ospedale privato<br>Ospedale universitario<br>Clinica privata<br>Servizio pubblico ambulatoriale<br>Servizio ambulatoriale privato<br>Servizio pubblico a domicilio<br>Servizio privato a domicilio
8. Quanto spesso tratti pazienti con frattura dell'anca?	Molto spesso (più di 10 pazienti al mese)<br>Spesso (tra 5 e 10 pazienti al mese)<br>Occasionalmente (tra 1 e 5 pazienti al mese)<br>Raramente (tra nessuno e 1 paziente al mese)
9. C'è un team multidisciplinare nel luogo in cui lavori che effettua la valutazione del paziente con una frattura del femore	Sì<br>No
10. In caso affermativo, alla domanda 9. Da chi è composto il team multidisciplinare?	Fisioterapista<br>Medico ortopedico<br>Medico fisiatra<br>Medico geriatra<br>Infermiera<br>Altro (specificare)
11. Che tipo di intervento chirurgico è più comune nella struttura in cui lavori?	Chiodo endomidollare<br>Protesi d'anca
12. Quale tipo di carico è più frequente dopo l'intervento chirurgico che hai selezionato nella domanda precedente?	Divieto di Carico<br>Carico parziale<br>Carico a tolleranza<br>Nessuna indicazione
13. Da chi ricevi l'indicazione di carico?	Medico ortopedico<br>Medico fisiatra<br>Medico geriatra<br>Altro (indicare quale)
14. Quanto pensi che il team multidisciplinare possa influenzare positivamente il recupero del paziente?	Molto<br>Abbastanza<br>Un po'<br>Inutile
15. Quanto pensi che un programma di mobilizzazione precoce (entro 48h) rispetto ad uno tardivo (oltre 48h) possa influire positivamente sul recupero del paziente con frattura del femore?	Molto<br>Abbastanza<br>Un po'<br>Inutile

16. Quanto pensi che un programma di riabilitazione intensiva rispetto ad uno non intensivo possa influire positivamente sul recupero del paziente con frattura del femore?	Molto<br>Abbastanza<br>Un po'<br>Inutile
17. Secondo lei, qual è il numero ottimale di sedute di fisioterapia in fase acuta per il paziente con frattura del femore?	Almeno una seduta al giorno<br>Una sessione a settimana<br>Una sessione al giorno<br>Tre sessioni a settimana<br>Due sessioni a settimana
18. Quanto dura una seduta media di fisioterapia per questo tipo di paziente in fase acuta (48-72H) nel luogo in cui lavori?	0-15 minuti<br>15-30 minuti<br>30-45 minuti<br>45 minuti - 1 ora<br>Oltre 1 ora
19. Dopo quanto tempo viene verticalizzato di solito il paziente post intervento chirurgico per frattura del femore?	Entro 24 ore<br>Tra 24 e 48 ore<br>Tra 48 e 72 ore<br>Oltre 72 ore
20. Che tipo di esercizio proponi più frequentemente a questi pazienti nelle prime 48-72h? (Inserire altre risposte se necessario)	Allenamento progressivo della forza muscolare<br>Esercizi con pesi<br>Allenamento dell'andatura<br>Altro (specificare)
21. È importante l'intervento di altre figure professionali per la mobilizzazione del paziente con frattura del femore in fase acuta?	Sì (specificare)<br>No
